# Histopathology and the inflammatory response of European perch, *Perca fluviatilis* muscle infected with *Eustrongylides* sp. (Nematoda)

**DOI:** 10.1186/s13071-015-0838-x

**Published:** 2015-04-15

**Authors:** Bahram S Dezfuli, Maurizio Manera, Massimo Lorenzoni, Flavio Pironi, Andrew P Shinn, Luisa Giari

**Affiliations:** Department of Life Sciences and Biotechnology, University of Ferrara, St. Borsari 46, 44121 Ferrara, Italy; Faculty of Biosciences, Agro-Alimentary and Environmental Technologies, University of Teramo, St. Crispi 212, I-64100 Teramo, Italy; Department of Cellular and Environmental Biology, University of Perugia, St. Elce di sotto 5, 06123 Perugia, Italy; Fish Vet Group Asia Limited, 99/386, Chaengwattana Building, Chaengwattana Rd., Kwaeng Toongsonghong, Khet Laksi, Bangkok 10210 Thailand

**Keywords:** Nematode larvae, Piscidin 3, PCNA, Macrophage aggregates, Mast cells, Fibroblasts

## Abstract

**Background:**

The European perch, *Perca fluviatilis* L. is a common paratenic host of dioctophymatid nematodes belonging to the genus *Eustrongylides.* In this host, once infected oligochaetes, which serve as the first intermediate host, are ingested, *Eustrongylides* migrates through the intestine and is frequently encountered within the musculature, free within the body cavity, or encapsulated on the viscera. The current study details the first Italian record of *Eustrongylides* sp. with larvae reported in the muscle of *P. fluviatilis*.

**Methods:**

Uninfected and nematode-infected muscle tissues of perch were fixed and prepared for histological evaluation and electron microscopy. Some sections were subjected to an indirect immunohistochemical method using anti-PCNA, anti-piscidin 3 and anti-piscidin 4 antibodies.

**Results:**

A total of 510 *P. fluviatilis* (TL range 15–25 cm) from Lake Trasimeno, Perugia were post-mortemed; 31 individuals had encysted nematode larvae within their musculature (1–2 worms fish^−1^). Histologically, larvae were surrounded by a capsule with an evident acute inflammatory reaction. Muscle degeneration and necrosis extending throughout the sarcoplasm, sarcolemmal basal lamina, endomysial connective tissue cells and capillaries was frequently observed. Within the encapsulating reaction, macrophage aggregates (MAs) were seen. Immunohistochemical staining with the proliferating cell nuclear antigen (PCNA) revealed numerous PCNA-positive cells within the thickness of the capsule and in the immediate vicinity surrounding *Eustrongylides* sp. larvae (*i.e.* fibroblasts and satellite cells), suggesting a host response had been initiated to repair the nematode-damaged muscle. Mast cells (MCs) staining positively for piscidin 3, were demonstrated for the first time in response to a muscle-infecting nematode. The piscidin 3 positive MC’s were seen principally in the periphery of the capsule surrounding the *Eustrongylides* sp. larva.

**Conclusions:**

A host tissue response to *Eustrongylides* sp. larvae infecting the musculature of *P. fluviatilis* was observed. Numerous fibroblasts, MAs and MCs were seen throughout the thick fibroconnectival layer of the capsule enclosing larvae. PCNA positive cells within the capsule suggest that host repair of nematode damaged muscle does occur, while the presence of the antimicrobial peptide piscidin 3 is shown for the first time. This is first report of *Eustrongylides* sp. in an Italian population of *P. fluviatilis*.

## Background

The European perch, *Perca fluviatilis* L., is widely distributed throughout the Palaearctic region and serves as a common paratenic host to the third or fourth stage larvae of several species of nematode belonging to the genus *Eustrongylides* Jägerskiöld, 1909 (Fam. Dioctophymatidae Railliet, 1915) [1]. *Eustrongylides* species have complex, indirect life-cycles involving aquatic oligochaetes, as the first intermediate hosts, fish, amphibians and/or reptiles as the second intermediate or paratenic hosts, and piscivorous birds as the definitive hosts. In birds, *Eustrongylides* spp. infect the outer surface of the ventriculus; nematode induced lesions both here and in abdominal cavity can lead to inflammation, bacterial peritonitis and septicaemia. Infections have been associated with epizootic episodes resulting in the significant mortality of a range of nestling ardeids (*i.e.* bitterns, egrets, herons *etc.*) and other wading birds [2-4]. In those fish species that serve as a paratenic host, *Eustrongylides* spp. migrate into the body and are frequently encountered within the skeletal musculature and only occasionally are found free within the body cavity or encapsulated on the viscera [1]. Infections of *Eustrongylides* sp. larvae within the ovaries of tank gobies, *Glossogobius giuris* F. Hamilton, 1822, have been reported to induce necrosis, resulting in a decrease in yolk formation, and, the disappearance of vitellogenic oocytes [5].

Although there are numerous literary accounts relating to the biosystematics, ecology, and zoogeography of fish nematodes [6], there are only a small number that detail the histopathology associated with their infection [7-11]. Infections within the livers of fish are perhaps the best and most extensively described [8,12,13], yet relatively few have reported on infections within fish host musculature [9,11,14,15]. Infections of *Philometroides paralichthydis* Moravec et de Buron 2006, for example, have been reported to impact on the locomotor performance leading to the complete atrophy of the fin inclinator muscles with consequential impairment to feeding efficiency in southern flounders, *Paralichthys lethostigma* D. S. Jordan et C. H. Gilbert, 1884 [9,16].

While the fish response to extraintestinal parasites in the formation of connectival capsules on the intestine and within the viscera for several parasite genera is well described [12,17,18], for muscle-invading nematodes our knowledge is scanty. For nematodes penetrating the muscles of mammals, e.g. *Trichinella* Railliet, 1895, there are different host responses to encapsulated and non-capsulated species of *Trichinella* [19]. In fish, the innate defence in response to helminth infection involves a variety of cells and these will be commented upon later in this account. A recent review by Kharraz *et al.* [20] also highlights the central role played by macrophages in the repair of skeletal muscle after acute damage. Macrophage aggregates in association with a range of parasitic infections in fish muscle have, for example, been reported upon with xenoma forming microsporidians like *Glugea anomala* (Moniez, 1887) Gurley, 1893 [21] and digenean metacercariae [22]. In addition to these, some oval-round shape cells, which resemble MCs, occurred within the periphery of the capsule; fish MCs possess cytochemical and distribution features that have led to the suggestion that they are analogous to mammalian MCs [23]. Fibroblasts also within the capsule, which are involved in the initiation of wound repair and regeneration, that surround the nematode larvae are evident [24], their cooperation with MCs in both fish and mammalian hosts leads to skeletal muscle repair [20,25,26]. Further to this, a range of antimicrobial peptides (AMPs), produced by vertebrate and invertebrate hosts, are a key factor in innate immunity [27]; in fish a common group of AMPs are the piscidins [28] and these have been isolated from MCs from a wide range of teleost taxa [29].

Changes in the expression of PCNA, a 36 kd protein involved in protein synthesis, can provide an early indication of changes in cell proliferation [30] and can be detected via immunohistochemical staining [31]. Alterations in the expression of PCNA has recently been applied to the field of fish parasitology [32,33] and to fish health [34,35], however, to date there are no known records regarding the expression of PCNA in parasite-infected fish muscle. In the current study of a nematode infecting the musculature of *P. fluviatilis*, the production of AMPs and the expression of PCNA is investigated in an attempt to provide further information regarding the host response. An emphasis will be placed on the role of MAs and fibroblasts as important components of the host’s innate immune system against helminth larvae and their involvement in muscle tissue regeneration will be commented upon.

*Perca fluviatilis* is an important commercial fishery and aquaculture species [36]. In 2012, according to FAO FishStatJ [37] and for which the latest figures are available, aquaculture ventures in 11 European states in 2012 produced 435 tons of *P. fluviatilis*; Italy was the fourth largest producer. Commercial fishing operations on Lake Trasimeno in the Province of Perugia in Central Italy, however, harvest c. 15 tons of *P. fluviatilis* p.a. and they noted a 6% of fillets, since 2011, infected with nematodes. This study in addition to providing more information on fish host responses to intra-muscular infections of nematodes, is also the first record of *Eustrongylides* sp. from fish muscle from Italy, a zoonotic species that may pose a public health risk to consumers. Humans who have consumed raw or undercooked fish infected with *Eustrongylides* have experienced gastritis of inflammation of the stomach and intestinal perforation requiring surgical removal of worms [38-40].

## Methods

### Sample collection and morphological identification of nematodes

A total of 510 *P. fluviatilis* (20.55 ± 6.44 cm, mean total length ± standard deviation; total length ranged from 15–25 cm) were collected from six sampling episodes conducted in Lake Trasimeno (43° 07’ 51.18” N; 12° 05’ 41.09” E) in the Province of Perugia in Central Italy throughout April to June 2014. On each sampling occasion, the fish were caught by gill net operated by professional fishermen belonging to the local fishing consortium. The live fish were then immediately transported to a holding tank within the Consortium’s facility; individual fish were systematically removed, euthanised using 125 mg L^−1^ MS222 (tricaine methanesulfonate, Sandoz, Basel, Switzerland) until opercular movements ceased; thereafter the spinal cord was severed and then the fish lengthed. Upon post mortem, the fish were first sexed before the gills and the viscera were removed and systematically screened, with the aid of a dissecting microscope, for the presence of helminths. The skeletal musculature of each specimen was methodically examined by removing entire blocks of muscle and removing thin (2–3 mm) slices and placing the individual tissue sections on a light box to assist in the visualisation of encysted nematodes. When nematodes were encountered, their exact position was recorded before 15 × 15 mm pieces of tissue that surrounded the nematode was excised and then fixed in chilled (i.e. 4°C) 10% neutral buffered formalin for 24 h. Thereafter, the fixed tissues were rinsed in several changes of 4°C, 70% ethanol and then stored in the same medium until they were processed for histology.

A random sub-sample of ten nematode specimens were removed from the fillets during the post-mortem evaluation and fixed in 70% ethanol. The larval nematodes were then sent to Dr Moravec at the Institute of Parasitology, Academy of Sciences of the Czech Republic, České Budĕjovice for identification.

### Histology and transmission electron microscopy

The fixed tissues were sequentially dehydrated through an alcohol series and then paraffin wax and embedded using a Shandon Citadel 2000 Tissue Processor (Shandon Citadel 2000, London, UK). After blocking out, 5 μm thick sections were taken from each tissue block and stained with haematoxylin and eosin (H&E), alcian blue/periodic acid Schiff (AB/PAS) and/or Masson’s trichrome.

In addition to the samples taken for conventional histological evaluation, a sub-sample (n = 7) of the nematode infected muscle tissues were prepared for electron microscopy. Each tissue specimen measuring up to 7 × 7 mm were excised and then fixed in chilled (i.e. 4°C), 2.5% glutaraldehyde solution in 0.1 M sodium cacodylate buffer, pH 7.3. After 2.5 h, the tissues were rinsed for 12 h in 0.1 M sodium cacodylate buffer containing 6% sucrose. The specimens were then post-fixed in 1% osmium tetroxide in the same buffer for 3 h, dehydrated through a graded acetone series, and then embedded in Epoxy resin (Durcupan ACM, Fluka, Buchs, Switzerland). Semi-thin sections (i.e. 1.5 μm) were cut on a Reichert Om U 2 ultramicrotome using glass knives and then stained with toluidine blue. Ultra-thin sections (90 nm) were cut with a diamond blade, stained with 4% uranyl acetate in 50% ethanol and Reynold’s lead citrate and examined using a Hitachi H-800 electron microscope. For comparative evaluation, muscle samples from 15 uninfected *P. fluviatilis* were processed alongside the nematode-infected tissue specimens for each technique.

### Immunohistochemistry

Two sections per wax block were taken from all nematode-infected hosts and from 15 uninfected hosts, and subjected to an indirect immunohistochemical method (peroxidase-anti-peroxidase immunocomplex) using a commercially available anti-PCNA antibody (PC10 sc-56 mouse monoclonal antibody, Santa Cruz Biotechnology, Inc.). After dewaxing in xylene and rehydrating through a graded alcohol series, the sections were treated for antigen retrieval in a citrate buffer (pH 8.0) for 20 min in a steam bath at 95°C; thereafter, the slides were left for 10 min to cool to room temperature (RT). Endogenous peroxidase activity and non-specific staining were blocked, respectively, in 3% H_2_O_2_ for 10 min and then in normal horse serum (1:20, Elite Mouse IgG Vectastain ABC Kit, Vector, Burlingame, USA) for 30 min. Sections were then incubated with the primary antibody (anti-PCNA diluted 1:500) for 2 h at RT. After washing with PBS, the slides were incubated for 30 min with biotinylated horse anti-mouse serum (Mouse IgG Vectastain ABC Kit, Vector) followed by avidin-conjugated horseradish peroxidase (Mouse IgG Vectastain ABC Kit, Vector). The sections were then developed using DAB (3,3′-diaminobenzidine 0.04% w/v in TBS 0.05 M, pH 7.4) and H_2_O_2_ (0.005%), rinsed and then counterstained with Harris’s haematoxylin. Non-immune mouse serum and diluent-only sections were used as negative controls.

Additional sections, i.e. two sections per tissue block from both infected and uninfected fish, were subjected to the IHC method using anti-piscidin 3 (anti-HAGR) and anti-piscidin 4 (anti-5.3-02-3A) antibodies. The two primary antibodies against piscidins were produced by a commercial laboratory (Bethyl Laboratories, Montgomery, Texas, USA) using the company’s standard procedures, which are detailed in Dezfuli *et al.* [41] and Corrales *et al.* [42]. Briefly, sections (5 μm) were dewaxed in xylene, rehydrated through a graded alcohol series, then endogenous peroxidase activity and non-specific staining were blocked in 3% H_2_O_2_ for 10 min and then in normal goat serum (1:20, Elite Rabbit IgG Vectastain ABC Kit, Vector, Burlingame, USA) for 30 min. After incubation with the primary antibodies (anti-HAGR diluted 1:400 and anti-5.3-02-3A 1:8000) for 3 h at RT, the sections were incubated for 30 min with a biotinylated goat anti-rabbit serum (Elite Rabbit IgG Vectastain ABC Kit, Vector), and then for 30 min with avidin-conjugated horseradish peroxidase (Elite Rabbit IgG Vectastain ABC Kit, Vector). The enzyme activity was detected using DAB. The sections were then dehydrated, counterstained with Harris’s haematoxylin. Non-immune serum and diluent-only sections were used as negative controls. Intestinal tissue from hybrid striped bass, *Morone saxatilis* (Walbaum, 1792) × *M. chrysops* (Rafinesque, 1820), was used as the positive control. The specificity of the reaction was confirmed by pre-absorption of each antiserum with the corresponding antigen.

All sections were examined and photographed using a Nikon Microscope ECLIPSE 80i.

## Results

### Sample collection and morphological identification of nematodes

**Figure 1:**
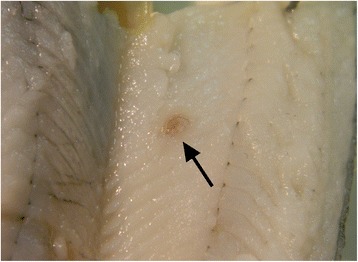
An encysted *Eustrongylides* sp. larva (arrow) within the epiaxial muscle of *Perca fluviatilis* L.

From a total of 510 specimens of *P. fluviatilis* from Lake Trasimeno that were post-mortemed and screened for nematode infections, 31 individuals (i.e. 6%) were found to harbour intra-muscular, encysted nematode larvae (1–2 worms fish^−1^; n = 50) (Figure 1). The vast majority of larvae were encountered within the epaxial muscle and were evident by their large size (c. 30 mm in length), by their red coloration and by the distortion their presence imposed on the musculature (Figure 1). A light box, however, for the candling of fillets was also used in the screening process to ensure that all larvae were detected. No nematode larvae within the gills, spleens, livers, kidneys, gonads or intestinal tracts were found.

The identity of the larval nematodes sent to the Institute of Parasitology, České Budĕjovice, Czech Republic were confirmed as *Eustrongylides* sp., as larvae belonging to this genus are not allocated to a species group unless identity can be confirmed by infection of the definitive host.

### Histology

**Figure 2:**
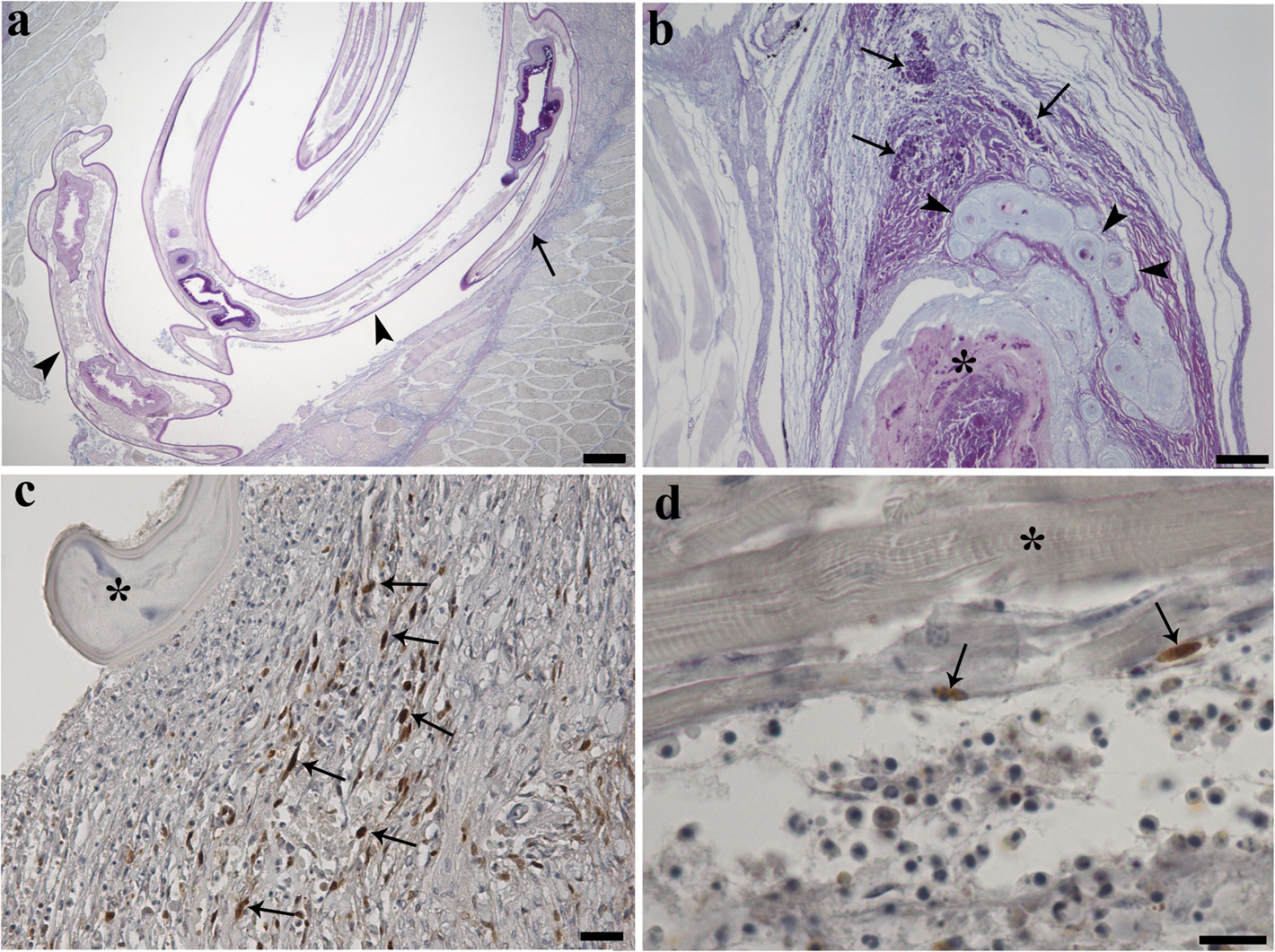
Alcian blue and periodic acid Schiff (PAS) stained histological sections through the skeletal muscle of *Perca fluviatilis* L. (**a**) Larval specimens of *Eustrongylides* sp. (arrow heads) within the muscle of *P. fluviatilis* where the nematode has replaced large portions of the skeletal muscle resulting in compression (arrow); scale bar = 200 μm. (**b**) PAS-positive macrophage aggregates (arrows) and multifocal fibro-epithelioid granulomata (arrow heads) are evident within the thickness of the capsule enclosing the *Eustrongylides* sp. larva. Note the PAS-positive necrotic material (asterisk); scale bar = 100 μm. (**c**) Histological section through nematode-infected muscle that has been stained with a PCNA-antibody. Positive fibroblasts (arrows) are scattered among the collagen fibres. Part of the nematode (asterisk) can be seen compressing the muscle tissues; scale bar = 20 μm. (**d**) PCNA-positive satellite cells (arrows) in the outer part of the myotubes (asterisk) can be seen; scale bar = 10 μm.

Figure 2a shows a histological section of *P. fluviatilis* musculature occupied by a specimen of *Eustrongylides* sp. Nematode larvae were surrounded by a capsule and an evident acute inflammatory response. At the sites of infection, the host muscle was necrotic and in a state of degeneration which extended through the sarcoplasm, sarcolemmal basal lamina, endomysial connective tissue cells and capillaries. At the extremities of the spindle-shaped capsule, numerous intensively PAS-positive MAs and multifocal fibro-epithelioid granulomatas were observed within the thickness of the capsule (Figure 2b). In some instances, PAS-positive necrotic material was also seen (Figure 2b).

### Immunohistochemistry

Immunohistochemical staining with the anti-PCNA antibody revealed numerous PCNA-positive cells within the thickness of the capsule, in the immediate vicinity surrounding the nematode larvae (Figure 2c), and scattered among the collagen fibres. Parallel studies with the transmission electron microscopy confirmed the identity of these positive cells as fibroblasts. In several sections of infected musculature, satellite cells in the outer part of the myotubes also stained positively with the anti-PCNA antibody (Figure 2e). Immunostaining of both infected and uninfected muscle with antibodies against the antimicrobial peptides piscidin 3 and piscidin 4 revealed a number of positive MCs. Only the MCs located in the periphery of the capsule surrounding the *Eustrongylides* sp. were positive to piscidin 3. These MCs were irregular in shape, possessed an eccentric, polar nucleus, and a cytoplasm containing membrane-bounded granules. No MCs positive for piscidin 4, however, were found.

### Transmission electron microscopy

**Figure 3:**
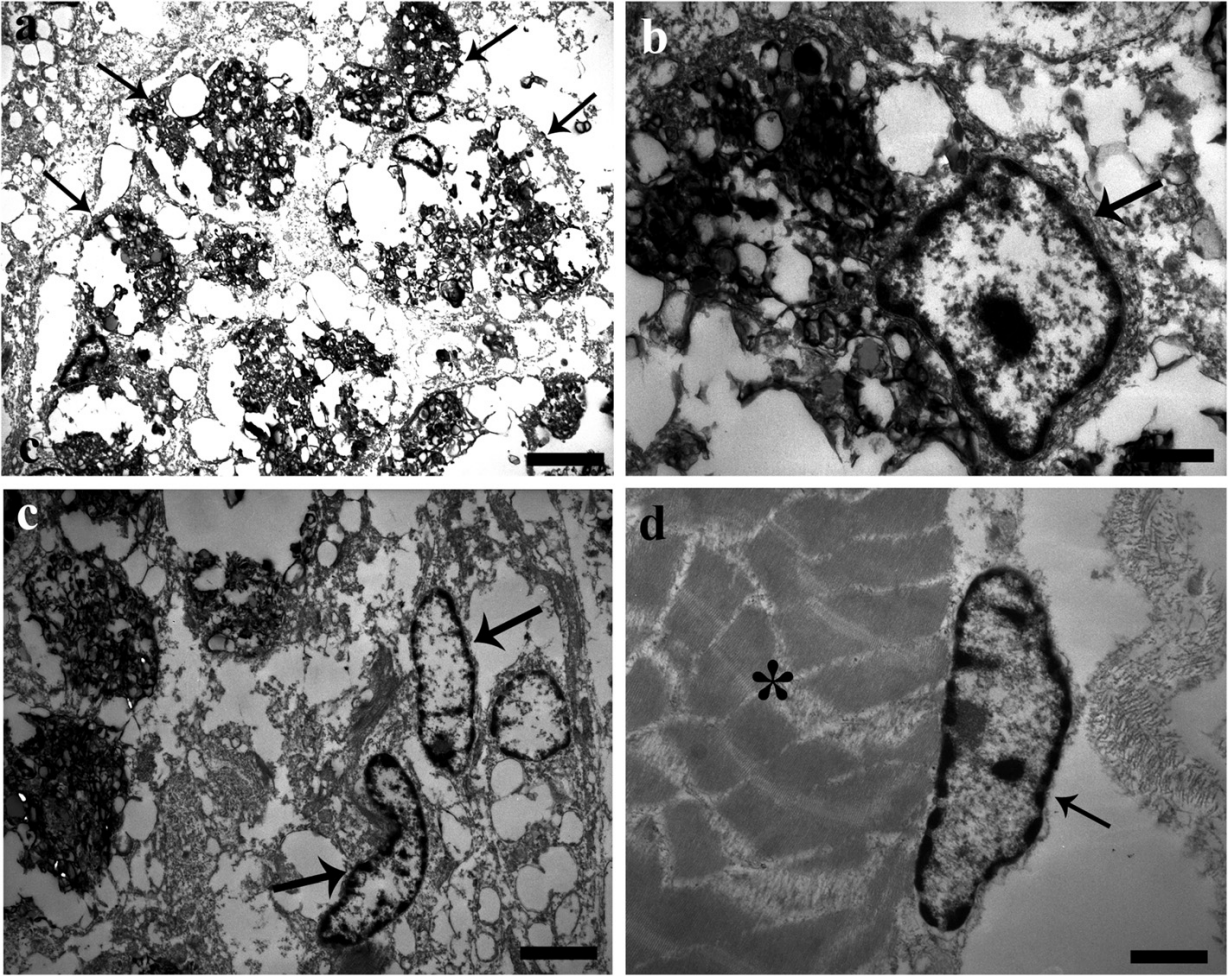
Transmission electron micrographs of *Eustrongylides-*infected *Perca fluviatilis* L. muscle. (**a**) Low magnification of the macrophages aggregates (MAs) in association with the *Eustrongylides* sp. larvae. The MAs appeared as group of large oval to round shaped cells (arrows) with a vacuolated and foamy appearance to their cytoplasm; scale bar = 3.3 μm. (**b**) Micrograph shows a macrophage aggregate with an eccentric polar nucleus (arrow) with marginal accumulations of chromatin. There are also cytoplasmic inclusions of various differing electron-densities, scale bar = 0.7 μm. (**c**) Two fibroblasts within the thickness of the capsule enclosing the larvae of the *Eustrongylides* sp., which are characterised by possessing elongated nuclei (arrows) and narrow cytoplasm, scale bar = 2.2 μm. (**d**) High magnification of a satellite cell beneath the basal lamina of the myofibres (asterisk) which possesses an elongated nucleus (arrow) and scarce-narrow cytoplasm; scale bar = 0.9 μm.

Transmission electron microscopy examination of the MAs in association with the nematode larvae revealed that they consist of a group of large oval to round cells (Figure 3a) with an eccentric polar nucleus with marked peripheral accumulations of chromatin (Figure 3b). The cytoplasm of these cells at low magnification appeared partially vacuolated and foamy (Figure 3a) and contained inclusions of various differing electron-densities that are consistent with phagosomes and phagolysosome containing lipids (i.e. those of the lowest osmiophilic content) and chromolipoids, principally ceroid (i.e. those of the highest osmiophilic content) (Figure 3b). Likewise, the fibroblasts distributed between the collagenous fibres possessed an elongated nucleus, accumulations of chromatin and a narrow cytoplasm containing no visible organelles (Figure 3c). Satellite cells either beneath the basal lamina of myofibres (Figure 3d) and/or free between the myotubes were also seen in the vicinity of nematode larvae; cells typically had an elongated nucleus within a narrow cytoplasm with no evident organelles.

## Discussion

Larval parasites within paratenic hosts rarely grow; likewise larval stages in intermediate hosts frequently stop growing before their growth is compromised by limited host resources and enter a stage of growth arrest. At this stage, the parasite may become encapsulated, which as an evolved strategy can reduce their mortality rate [43]. Encapsulation, however, is mutual adaptation between the host’s immune response and parasite, reaching a strategic compromise between the two to ensure the survival of both species [19]. The various life strategies employed by parasite species can influence different aspects of host physiology and so activate different immunological pathways [44]. The manner in which mammalian muscle responds to encapsulating species of nematode like *Trichinella spiralis* (Owen, 1835), for example, which elicits an inflammatory reaction, is different to that of non-encapsulating species of nematode like *Trichinella pseudospiralis* Garkavi, 1972, [19]. During infection with *T. pseudospiralis*, a lower number or total absence of infiltrating cells were noticed [19]. *Eustrongylides* sp. as encapsulating species of nematode also induces an intense muscle inflammatory response.

The encapsulation and host tissue response to parasite stages within the digestive tract and associated organs can lead to the formation of granulomas [36] – a chronic inflammatory lesion [45], which in fish involves the activity of macrophages, lymphocytes, granulocytes and fibroblasts depending on the eliciting agents and host species [22,46]. The lesion or granuloma is typically organised in concentric cellular layers, the central part being formed by macrophages encircled by a collar of other, different inflammatory cells [46,47]. In fish, the innate defences responding to helminth infection commonly involve a variety of cells such as neutrophils and fibroblasts [48-50], macrophages [18,51,52], MAs or melano-macrophage centres [53-55] and MCs [10,22,56,57]. In rainbow trout, *Oncorhynchus mykiss* (Walbaum, 1792), the relationship between MCs and fibroblasts has been described with fibroblasts influencing MC motility [26,58,59]. In mammals [20,25,60] and in fish [26,59,61], several lines of evidence suggest that MCs are involved in fibrotic processes and in tissue remodelling. In the current study, no neutrophils were encountered at the sites of nematode infection and the reason(s) for their absence is, as yet, unknown and is open to conjecture. Likewise, no cellular host reaction was seen in the muscles of southern flounders, *P. lethostigma,* infected by two species of philometrid nematode [9].

In the current study, MAs were the dominant host immune elements seen in close proximity to the nematode larva. The presence of MAs in close association with other parasitic infections in fish have been reported and include: microsporidian xenomas within the musculature [21], systemic infections of digenean metacercariae [22], cestodes [32] and nematodes within the liver [8].

The nature and role of MAs in fish pathology has been comprehensively reviewed [54] and their proliferation in association with a range of physiological and pathological factors including aging, starvation, infectious disease, and chemical exposure has been documented [62-64]. The observations from the current study, however, lend support to the view of Vogelbein *et al.* [62] in that MAs may be linked to parasite infections and, in all likelihood, represent an inflammatory response that is different from the typical granulomatous reaction. Considering the significant number of MAs that were seen, one possible interpretation is that given the large size of the *Eustrongylides* sp. larvae, the parasites occupy and substitute a marked proportion of the host muscle elements and induce an extensive degeneration of the myofibres. Numerous MAs, therefore, are necessary to engulf the residue from the damaged myofibres. Based on the current study, it appears that an infection of *Eustrongylides* sp. in *P. fluviatilis* preferentially induces the recruitment of MAs and fibroblasts, and, to a lesser degree, that of other immune cells.

The oval to round shaped cell observed at the periphery of the capsule, which responded positively to the piscidin 3 antibody, were subsequently confirmed to be MCs. Piscidins have potent, broad-spectrum antimicrobial activity against a broad spectrum of viral, bacterial, fungal and parasite species [65-67]. Piscidin 3 has also been reported in MCs in the gills of European seabass, *Dicentrarchus labrax* (L.), infected with *Diplectanum aequans* (Wagener, 1857) [41] and gilthead seabream, *Sparus aurata* L., infected *Ergasilus* sp. [68], while piscidin 3 and 4 positive MCs have been seen in the liver and in the intestine of *P. fluviatilis* infected with the cestode *Triaenophorus nodulosus* (Pallas, 1781) [32] and the acanthocephalan *Acanthocephalus lucii* (Müller, 1776) [57]. Corrales *et al.* [69] observed that piscidin 4 in the gill MCs of hybrid striped bass, *M. saxatilis* × *M. chrysops,* were significantly lower in ectoparasite-infected gills, suggesting that piscidin 4 is significantly down-regulated during this parasitosis. In the present study, piscidin 4 expression in muscle MCs was absent. These two studies suggest that parasites may actively modulate AMP expression.

PCNA has been reported from a number of different organs in fish [70,71]. An increase in the expression of PCNA is widely accepted as a marker of proliferation associated with the development of neoplastic tissue [31,72,73]. An increase in PCNA labelling, therefore, signals marked increases in the rate of cellular division and yet limited information exists regarding the expression of PCNA in infected fish tissues (e.g., [33]). This study set to determine whether PCNA-positive cells were present within the nematode-infected muscles of *P. fluviatilis.* The study was able to demonstrate the presence of numerous proliferating cells in the capsule surrounding the nematode larvae; no positive cells were found in uninfected host muscles. The findings of this study represent the first record of PCNA-positive cells within the muscles of a teleost fish infected by an endoparasitic worm. The capacity of muscles to regenerate relies primarily on a specific population of normally quiescent muscle stem cells, named satellite cells due to their position and intimate association with muscle [20]. Like satellite cells, resident fibroblasts proliferate and migrate to the site of muscle damage where they function in close proximity to satellite cells and regenerating myofibres [20]. The abundant presence of both these cells at the site of nematode infection in the muscles of *P. fluviatilis* is in agreement with the hypothesis that host muscle responds to the injury caused by *Eustrongylides* sp. larvae and attempts to repair the damage.

## Conclusions

The post-mortem examination of 510 *P. fluviatilis* found that 31 specimens were infected with the larvae of a dioctophymatid nematode, subsequently identified as *Eustrongylides* sp. This represents the first record of this zoonotic nematode in Italy and may pose a public health risk to consumers. This encapsulating species of nematode induces an intense inflammatory response in fish muscle, involving fibroblasts, MCs and MAs, which were the dominant host immune elements observed.

## Abbreviations

AB/PAS: Alcian blue/periodic acid Schiff
AMPs: Antimicrobial peptides
DAB: Diaminobenzidine
H&E: Haematoxylin and eosin
IHC: Immunohistochemistry
MAs: Macrophage aggregates
MCs: Mast cells
P3: Piscidin 3
P4: Piscidin 4
PAS: Periodic acid Schiff
PCNA: Proliferating cell nuclear antigen
PBS: Phosphate buffered saline
RT: Room temperature
TBS: Tris buffered saline
